# Multislice helical computed tomography imaging diagnosis and surgical treatment of primary tracheal tumor in cardiothoracic surgery

**DOI:** 10.3389/fonc.2024.1376228

**Published:** 2024-05-27

**Authors:** Jun Rui, Yu Lian

**Affiliations:** Department of Cardiothoracic Surgery, Wuxi Second People’s Hospital, Wuxi, Jiangsu, China

**Keywords:** MSCT, CTVE, imaging diagnosis, primary tracheal tumor, tomography imaging

## Abstract

**Objective:**

it aimed to explore the value of multislice helical computed tomography (MSCT) in the diagnosis and surgical treatment of primary tracheal tumors.

**Methods:**

64 patients with the primary tracheal tumor who were diagnosed in Wuxi Second People’s Hospital from March 2020 to March 2021 were selected as the research objects. MSCT imaging was performed on all patients, and suitable surgical methods. The pathological results were compared with original CT, CT virtual endoscopy (CTVE), and Comparisons were made using CT three-dimensional reconstruction images to evaluate the accuracy of MSCT diagnosis. Parameters such as postoperative complications and survival rates were observed to assess surgical effectiveness and safety.

**Results:**

Compared with original CT images (70%, 72%, 70%), the diagnostic accuracy of VR images (80%, 80%, 80%), MPVR images (85%, 90%, 92%), and CTVE images (100%, 100%, 100%) was remarkably improved (P<0.05). The three-year survival rate of patients with smooth muscle tumors, malignant tumors, salivary gland adenoma, papillary tumors, and inflammatory polyp was markedly lower than that of the one-year survival rate, with a significant difference (P<0.05). The incidence of postoperative complications was 14.1%, with three cases resulting in complication-related deaths.

**Conclusion:**

the diagnostic accuracy of MSCT imaging of primary tracheal tumor was high. The diagnostic accuracy of CTVE was higher than that of VR and MPVR. Besides, surgical treatment of primary tracheal tumor had a substantial effect, with no serious postoperative complications.

## Introduction

The primary tracheal tumor is a tumor originating in the trachea, which has different growth trends due to the different benign and malignant factors and potentially poses a threat to the surrounding structures such as lungs, esophagus, and throat (1). The disease has a low incidence but a high rate of malignancy that is up to 95 percent according to some investigations. According to the degree of malignancy, primary tracheal tumors can be classified into malignant, low malignant, and benign (2). The main symptom of the disease is a chronic cough. In addition, patients also appear dyspnea, wheezing sound, hemoptysis, chest pain, hoarseness, and difficulty swallowing. The cause of the disease isn’t entirely clear (3, 4). Genetic factors play a crucial role in the development of the disease. Radiation, exposure to toxic and harmful chemicals, and chronic diseases of the trachea possibly are also the causes. People who have a family member with the disease as well as those who have a smoking habit, are at increased risk (5).

The main hazards of primary tracheal tumors include the blockage of the trachea, compression of the esophagus and surrounding blood vessels, and serious effects on breathing, swallowing, and blood supply to the head and neck. In severe cases, it causes direct death (6). Currently, surgery is the main treatment method for primary tracheal tumors, including endoscopic tumor resection, tracheal resection, tumor resection with tracheal defect repair, tumor resection with tracheal regrafting, and tumor resection with artificial tracheal replacement (7). Besides, it can be combined with chemotherapy, radiotherapy, and supportive treatment such as ventilator ventilation. With early and effective surgical treatment, it is possible to be completely cured. After the surgical treatment, some patients have tracheal pain, phlegm, dysphagia, and other sequelae, but the degree is usually mild (8, 9). Consequently, early detection and treatment of primary tracheal tumors are very important for clinical diagnosis and treatment.

At present, the clinical diagnostic means for primary tracheal tumors include physical examination, blood examination, X-ray, CT, magnetic resonance imaging (MRI), tracheography, and pathological biopsy, which often requires a combination of various results for diagnosis (10, 11). The pathological biopsy is the gold standard for determining primary tracheal tumor, which is the most precise examination. Endoscopy is used in most cases. After partial tumor tissue is extracted, pathological sections are made and analyzed to determine the type and degree of malignancy of the tumor (12). Cervicothoracic CT can be adopted to carefully determine the structure and location of tracheal tumors as well as the changes in the surrounding bones to determine whether there is bone metastasis (13). With the rapid development of microelectronics and computer technology, the basic theory and setup of CT have been improved and updated. Multislice helical CT (MSCT) developed in 1997 is an imaging system with a multi-row wide detector structure and single exposure of spherical tube that can simultaneously obtain image data of multiple layers (generally 4, 8, and 16 layers) (14).

Compared with single-slice helical CT (SSCT), MSCT has multiple data acquisition channels in addition to the multi-row detector structure on the Z-axis. Besides, the calculation methods used in image reconstruction are also different. MSCT is mainly improved in the scanning rack, detector, data acquisition system (DAS), image reconstruction system, and computer system (15). The isotropic imaging is adopted for musculoskeletal inspection, the special circumstances of multidirectional reconstruction CT angiography of the spinal cord, the large scope and multi-temporal study, CT angiography, cardiac assessment, cerebral perfusion imaging, the examinations of the patients with large size, the evaluation of patients with acute chest pain or shortness of breath, the artificial endoscope, and retrospective image fusion for thin layer scanning (16, 17).

At present, there are some studies on the diagnostic effect of MSCT imaging for primary tracheal tumors. Nevertheless, the MSCT imaging of primary tracheal tumors, CT virtual endoscopy (CTVE), volume rendering of primary tracheal tumor (VR), multiple planar reformations (MPR), multiplanar volumetric reconstructions (MPVR), and other CT image post-processing methods are combined to evaluate the comprehensive diagnostic effect of MSCT images and various image post-processing methods, which hasn’t been performed. Moreover, there are few studies on the combination of MSCT imaging diagnosis and surgical treatment of primary tracheal tumors. Hence, it was hoped to explore this issue in depth.

The innovation of this work lay in the analysis of MSCT image features of patients with different types of primary tracheal tumors and the comparative analysis of different MSCT image post-processing methods. Furthermore, the results of surgical treatment for these diseases were quantified to comprehensively evaluate MSCT imaging and surgical treatment for the diagnosis of primary tracheal tumor. It was hoped to provide some theoretical reference for the imaging diagnosis and surgical treatment of primary tracheal tumors.

## Materials and methods

### Research objects

64 patients with the primary tracheal tumor who were diagnosed in Wuxi Second People’s Hospital from March 2020 to March 2021 were selected as the research objects. Their ages ranged from 32 to 84 years old, with an average age of (59.86 ± 11.25) years old. The confirmed history was from 1 to 6 months.

The inclusion criteria were as follows. I. Patients diagnosed with the primary tracheal tumor; II. Patients who underwent the surgical treatment; III. Patients with complete medical records and who signed the informed consent. The exclusion criteria were as follows. I. Patients with other serious organ diseases; II. Patients with severe hepatic and renal insufficiency; III. Patients with disorders of consciousness or mental disorders.

All procedures of this experiment were approved by the ethics committee of Wuxi Second People’s Hospital (Approval Number: ****, Date **.), and all patients signed the informed consent.

### Equipment of MSCT and scanning parameters

The GE LightSpeed 64-slice spiral CT scanner was used in this experiment. **Table 1** showed the scanning parameters.

**Table 1:** Scanning parameters of CT.

Items	Parameter Values
Scanning Slice Thickness	7mm
Reconstruction Slice Thickness	0.625mm
Reconstruction Interval	0.625mm
Reconstruction Algorithms	Lung Algorithm and Standard Algorithm
Scanning Field of Vision	50cm
Pitch	0.516

The enhanced scanning scheme adopted was using the 70–90mL non-ionic iodine contrast agent at an injection rate of 3–5mL/s for intravenous infusion. Then, scans were performed at 20 to 25 seconds in the arterial phase and 90 seconds in the venous phase.

### MSCT scanning and image evaluation

**Figure 1:**
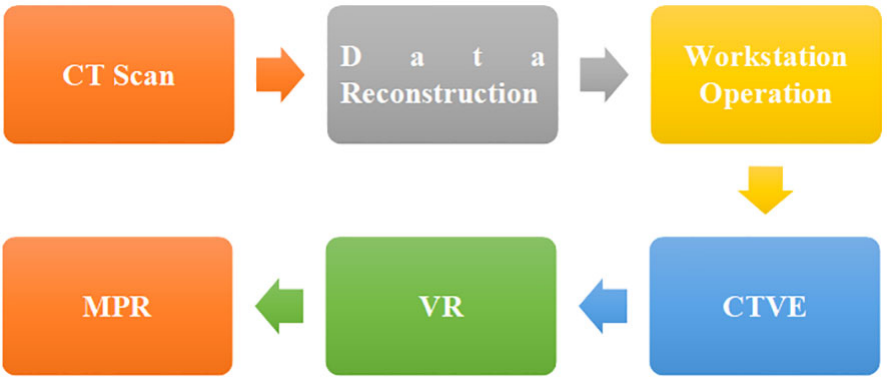
Flow diagram of MSCT image processing.

The original data obtained by CT instrument scanning was reconstructed and sent to the workstation for operation. CT virtual endoscopy (CTVE), volume rendering (VR), and multiple planar reformations (MPR) (18, 19) were adopted for the post-processing of CT images (**Figure 1**).

MPR was used to collect the transverse, sagittal, coronal, and oblique images of patients’ lesion areas, which were combined with the multi-window width and window level techniques to achieve centralized observation of the lesion area (20). VR could intuitively display the pleural depression of patients’ lesions as well as the relationship with blood vessels and airways (21).

All the MSCT image data were judged by 3 physicians with over 4 years of experience in chest CT diagnosis. All evaluations and judgments were made when the patient’s clinical data and pathologic type were unknown. The signs of MSCT image analysis included the shape, size, location, edge shape, characteristics of the tumor-lung surface, and adjacent structure and internal structure of tracheal tumor lesions.

The diagnostic criteria of tumor morphology, the extent of long axis stenosis, and the extent of involvement for MSCT imaging of the primary tracheal tumors were as follows. I. The primary tracheal tumors included the intraluminal narrow basal nodule, the intraluminal wide basal nodule, the infiltrative growth along the duct wall, the intraluminal and extraluminal mass, and the tracheal ridge mass; II. Lumen stenosis of tracheal tumors was classified into mild, moderate, and severe stenosis; III. The extent of wall involvement of tracheal tumors included<10mm, 11–50mm, and >51mm.

### Surgical treatment of primary tracheal tumors

Surgical approaches for patients with primary tracheal tumors included the cervical collar incision, median sternal split incision, and posterior-lateral thoracic incision. For the cervical segment and above the aortic arch, a cervical collar incision was selected, and the median of the upper sternum segment was split according to the situation, while a posterolateral incision was selected if the tumor occurred in the thoracic trachea. For a small amount of air leakage at the anastomotic site during operation, it was treated with adding the needles or covered with the pleura plus biological glue (Medical biological protein glue, National Medical Products Administration (NMPA) approval number S20120008, produced by Zhejiang Sailine Pharmaceutical Technology Co., Ltd.), and the sealing effect was satisfactory. The anastomosis was routinely covered with the nearby mediastinal pleura or pericardium, with special attention to separating it from the nearby blood vessels to prevent serious consequences caused by the friction damage to blood vessels. After the tracheal tumor was resected, For the tracheal reconstruction, it was ideal to remove the anastomosis at the back end. If there was the estimated residue, the silver clip was placed locally for marking. After surgery, radiotherapy and chemotherapy were supplemented.

The surgical method of primary tracheal tumor was carried out according to the clinical standard surgical method (18). When the surgery was completed, the fiber bronchoscope was used to examine the anastomosis before the tracheal intubation was removed.

### Postoperative observation index

Patients’ recovery after surgery was recorded and assessed through follow-up surveys ranging from 3 months to 6 years. The complications and deaths caused by the primary tracheal surgery were also recorded. The collected data were summarized and analyzed. Equation 1 showed the calculation of the incidence of the complications. Equation 2 showed how the mortality due to complications was calculated. Equations 3–5 showed the calculation of the one-year survival rate, the three-year survival rate, and the five-year survival rate, respectively.


(1) $$Q=\frac{\text{A}}{\text{N}}\times 100\%$$

(2) $$W=\frac{\text{a}}{\text{N}}\times 100\%$$

(3) $$E=\frac{\text{Z}}{\text{N}}\times 100\%$$

(4) $$R=\frac{\text{X}}{\text{N}}\times 100\%$$

(5) $$T=\frac{\text{C}}{\text{N}}\times 100\%$$

In Equations 1–5, Q represented the incidence of complications, A represented the number of patients with complications, N represented the total number of patients, W represented complications induced by death, and a represented complications induced death toll. E expressed the one-year survival rate, and Z expressed the number of survivals in one year after surgery. R presented the three-year survival rate, and X presented the number of survivals in three years after surgery. T presented the five-year survival rate, and C presented the number of survivals in five years after surgery.

### Statistical methods

SPSS 19.0 was employed for data statistics and analysis. Mean ± standard deviation (x ± s) was how measurement data were expressed, and the t test was used to compare the mean between groups. Percentage (%) was how count data were expressed, and the data was tested by χ2 test. The difference was statistically considerable with P<0.05.

## Results

### General data of patients

**Figure 2:**
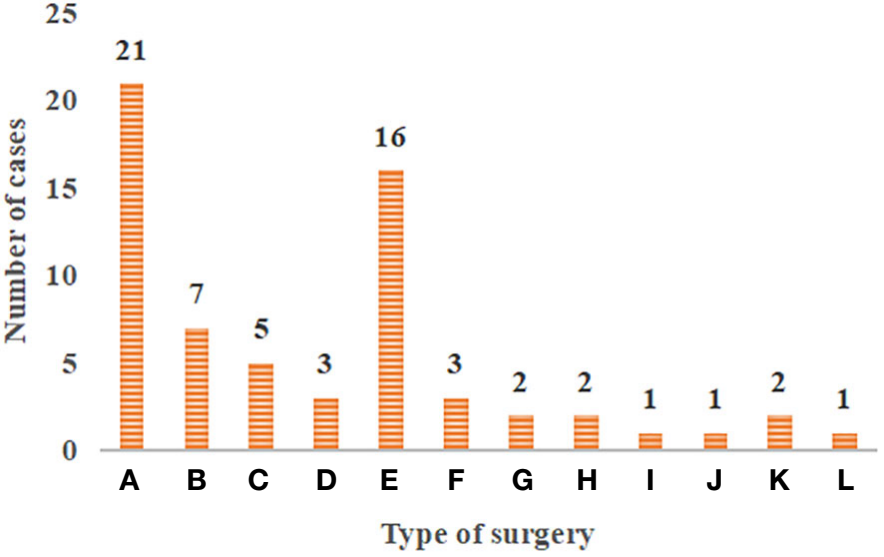
Distribution of surgical methods in patients with primary tracheal tumors. **(A–L)** The resection and reconstruction of the tracheal ridge, the resection and reconstruction of cervical tracheal tumor and vertical hemilaryngectomy, the resection and reconstruction of hemilaryngectomy, the complete resection of tracheal sleeve-shape with end-to-end anastomosis, local tracheal tumor resection, cervical tracheal resection, esophageal myotomy, tracheal resection and ostomy, the carina curettage, partial thyroidectomy, the simple tracheal resection and ostomy, and the cervical part of the trachea and total laryngectomy.

**Figure 3:**
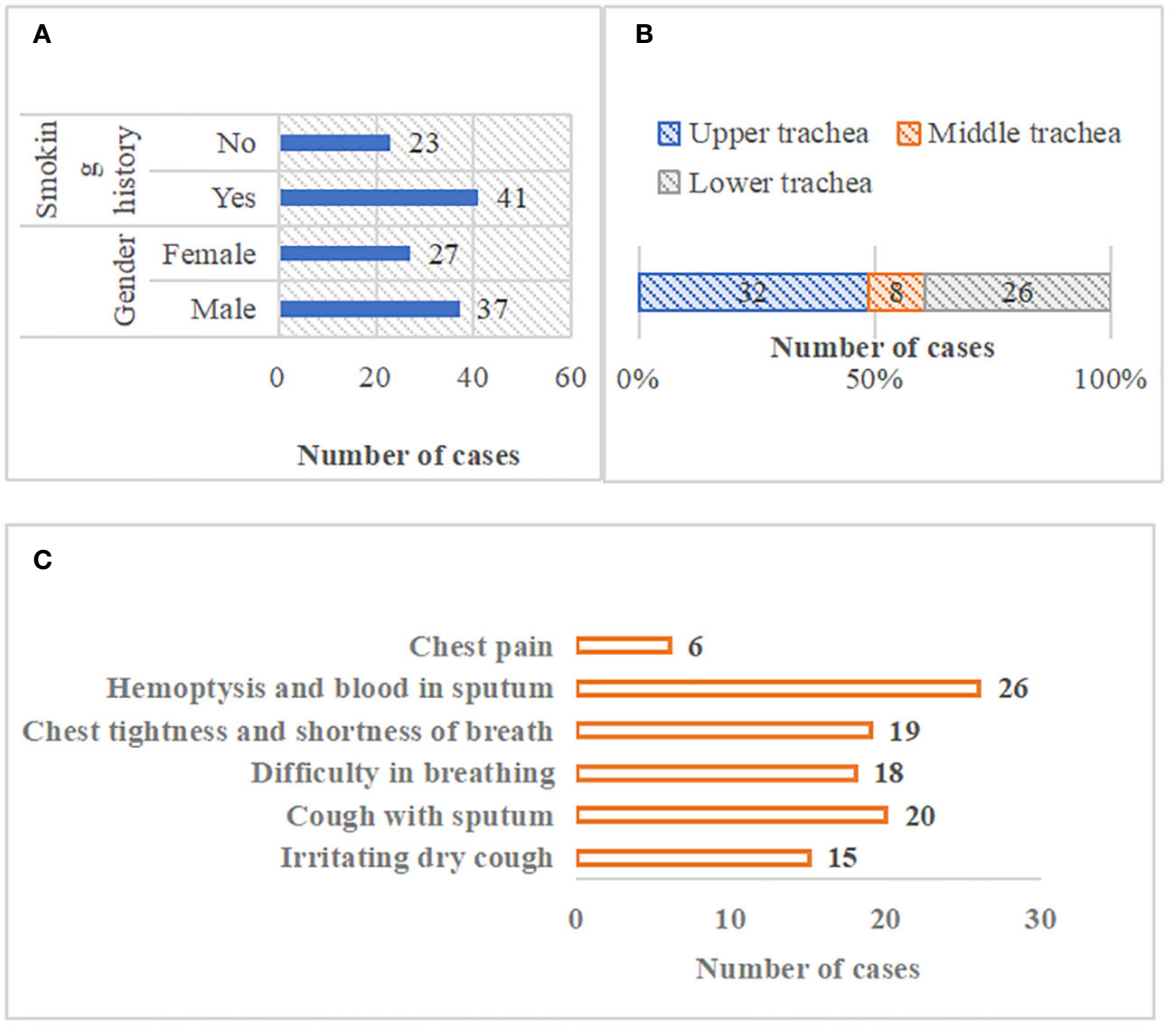
General data of patients. **(A)** patients’ gender and smoking history; **(B)** location distribution of tracheal tumor lesions; **(C)** clinical symptom distribution of patients).

**Figure 2** showed the general data of patients with primary tracheal tumors. There were 37 male patients and 27 female patients, 41 of which had smoking habits. There were 32 patients with the tracheal tumor lesions located in the upper trachea, 8 with that in the middle trachea, and 26 with that in the lower trachea. In terms of clinical symptoms, there were 15 cases of irritating dry cough, 20 of cough with sputum, 18 of difficulty in breathing, 19 of the chest tightness and shortness of breath, 26 of hemoptysis and blood in sputum, and 6 of chest pain (**Figure 3**).

### Surgical treatment methods

**Figure 4:**
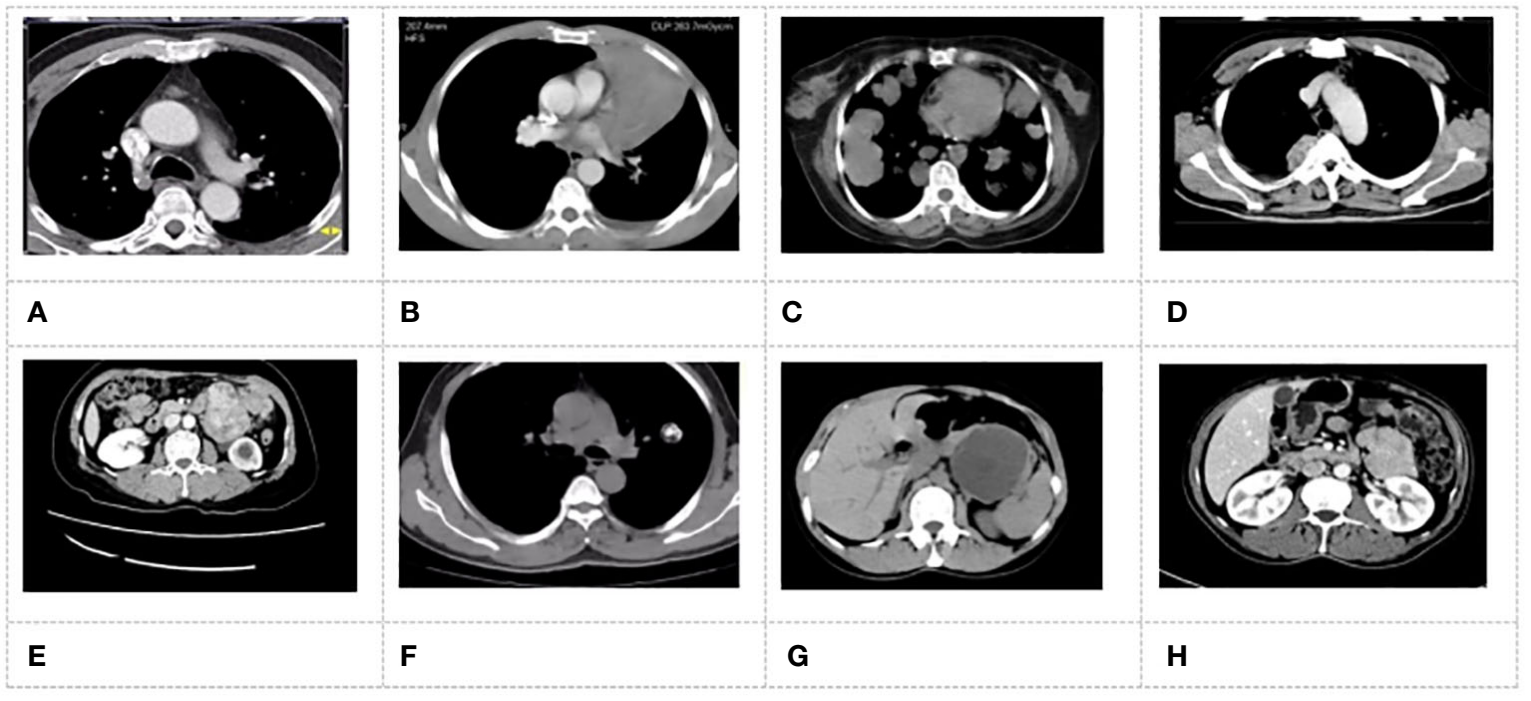
MSCT images of patients. **(A)** patient with adenocarcinoma; **(B)** patient with squamous carcinoma; **(C)** patient with adenoid cystic carcinoma; **(D)** patient with carcinoid tumor; **(E)** patient with smooth muscle tumor; **(F)** patient with malignant tumor; **(G)** patient with Sali vary gland adenoma; **(H)** patient with papillary tumor.

**Figure 2** showed the distribution of surgical methods in patients with primary tracheal tumors. In **Figure 4**, the surgical resection methods included the complete resection of various types of molding, the complete resection of the end-to-end anastomosis, and simple resection. For the complete resection of various types of molding, there were 21 cases of the resection and reconstruction of the tracheal ridge, 7 cases of the resection and reconstruction of cervical tracheal tumor and vertical hemilaryngectomy, 5 cases of the resection and reconstruction of hemilaryngectomy, and 3 cases of the complete resection of tracheal sleeve-shape with end-to-end anastomosis. As for the simple resection, there were 16 cases of local tracheal tumor resection, 3 cases of cervical tracheal resection, 2 cases of esophageal myotomy, 2 cases of tracheal resection and ostomy, 1 case of the carina curettage, 1 case of partial thyroidectomy, 2 cases of the simple tracheal resection and ostomy, and 1 case of the cervical part of the trachea and total laryngectomy.

### MSCT imaging results of patients

**Figure 4** showed the MSCT images of the patients with primary tracheal tumors. In **Figure 4**, adenoma MSCT was mainly manifested as a round nodular tumor protruding into the lumen, with a smooth edge and uniform density. The narrow base was connected with the tube wall without incrassation. The MSCT of carcinoid tumors and salivary gland carcinoma showed lobulated nodules, and there were destruction and displacement of trachea cartilage to a certain extent and the incrassation of the tracheal wall at the base. Adenoid cystic carcinoma tumors infiltrated along the long axis of the tracheal wall, which resulted in varying degrees of wall thickening and lumen narrowing. Squamous carcinoma was characterized by both intraluminal and extraluminal expansion, in which the extraluminal growth was dominant. Malignant squamous carcinoma presented the saddle-shaped swelling, with the narrow proximal bronchus on both sides, the uneven tumor surface, and mild lobulation.

### Patient’s tumor tissue type

**Figure 5:**
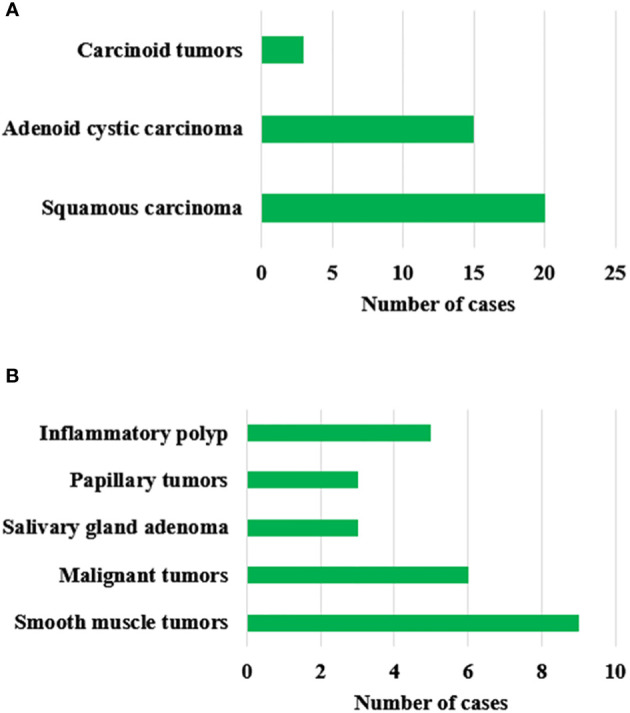
Postoperative pathological results of patients with primary tracheal tumors. **(A)** represents malignant tumors; **(B)** represents benign tumors.

**Figure 5** showed the postoperative pathological results of patients with primary tracheal tumors. In **Figure 5**, according to the postoperative pathologic examination of the enrolled 64 patients, there were 38 patients with malignant tumors and 26 with benign tumors. There were 20 cases of squamous carcinoma, 15 cases of adenoid cystic carcinoma, 3 cases of carcinoid tumors, 9 cases of smooth muscle tumors, 6 cases of malignant tumors, 3 cases of salivary gland adenoma, 3 cases of papillary tumors, and 5 cases of the inflammatory polyp.

### Comparison of pathological results and MSCT results

**Figure 6:**
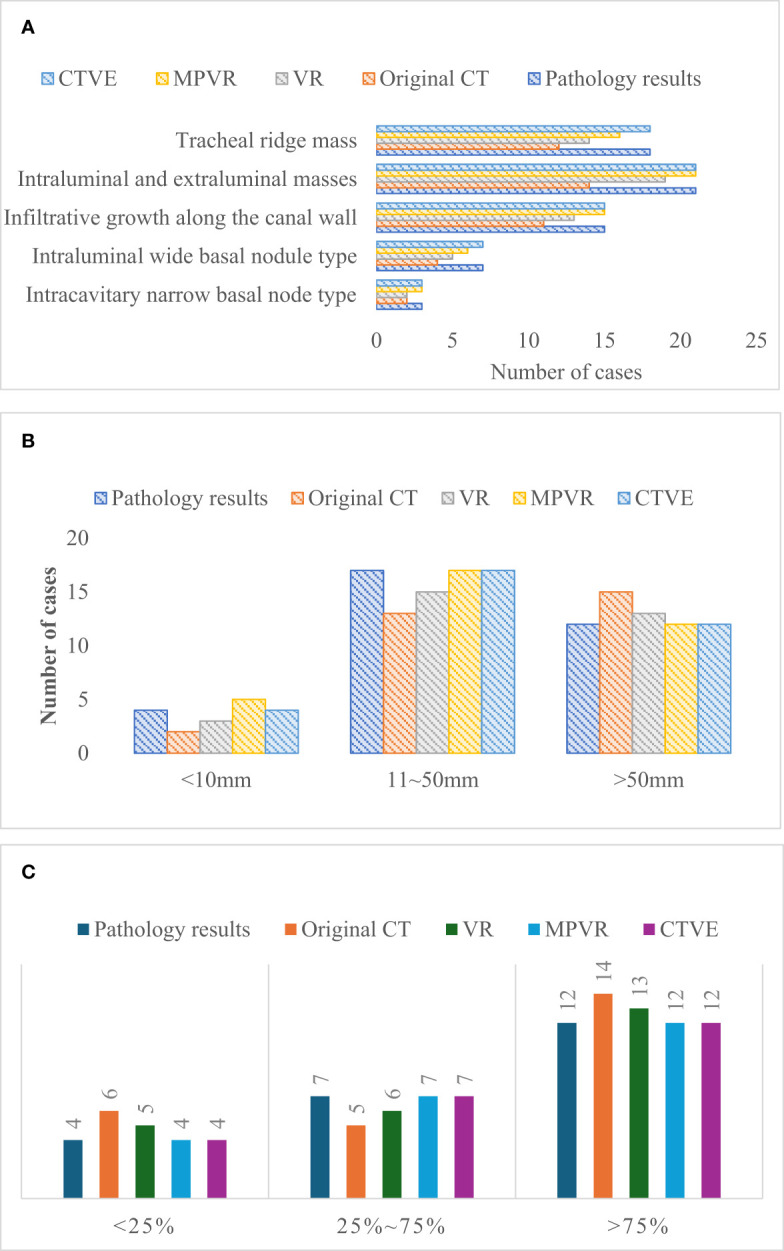
Comparison of the pathological results and MSCT results. **(A)** comparison of pathological results and MSCT results of tumor morphology; **(B)** comparison of pathological results and MSCT results of extent of long-axis involvement of duct wall; **(C)** comparison of pathological results and MSCT results of the extent of wall stenosis of tumor morphology.

**Figure 6** showed the comparison of the pathological results and MSCT results of patients. In terms of tumor morphology, there were 3 cases of the intraluminal narrow basal nodule, 7 cases of the intraluminal wide basal nodule, 15 cases of infiltrative growth along the duct wall, 21 cases of intraluminal and extraluminal mass, and 18 cases of tracheal ridge mass. The extent of long-axis of involvement of duct wall was<10mm in 4 cases, that was between 10 and 50mm in 17 cases, and that was > 50mm in 12 cases. Besides, in terms of the degree of wall stenosis, 4 cases had the stenosis degree below 25%, 7 cases between 25% and 75%, and 12 cases above 75%.

### Evaluation of diagnostic accuracy of patients’ MSCT images

**Figure 7:**
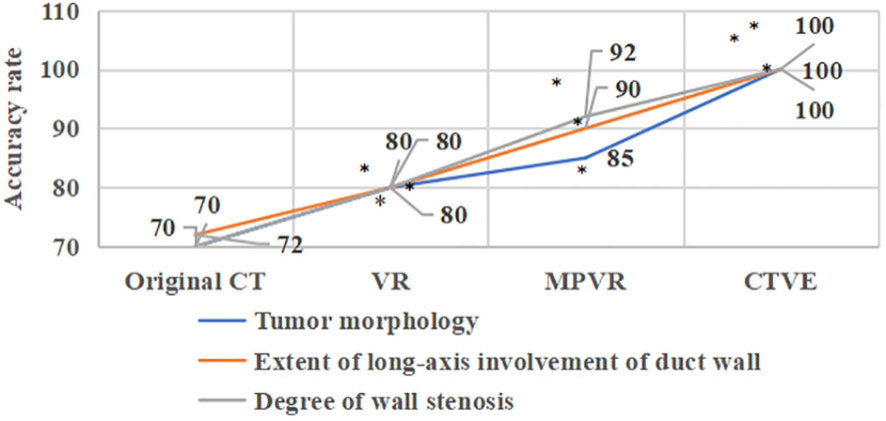
Comparison of the diagnostic accuracy of patients’ MSCT images; * meant that compared with the diagnostic accuracy of the original CT images, P<0.05.

**Figure 7** showed the comparison of diagnostic accuracy of patients’ MSCT images. In **Figure 7**, the diagnostic accuracy of tumor morphology, the extent of long-axis involvement of duct wall, and degree of wall stenosis in the original CT images of patients with primary tracheal tumors were 70%, 72%, and 70%, respectively. The diagnostic accuracy of tumor morphology, the extent of the long-axis involvement of the duct wall, and the degree of wall stenosis in the VR images were 80%, 80%, and 80% respectively. The diagnostic accuracy of tumor morphology, the extent of long-axis involvement of duct wall, and degree of wall stenosis in the MPVR images were 85%, 90%, and 92%, respectively, and those in the CTVE images were all 100%. Compared with the original CT images, the diagnostic accuracy of VR images, MPVR images, and CTVE images was greatly improved, with significant differences (P<0.05).

### Postoperative survival

**Figure 8:**
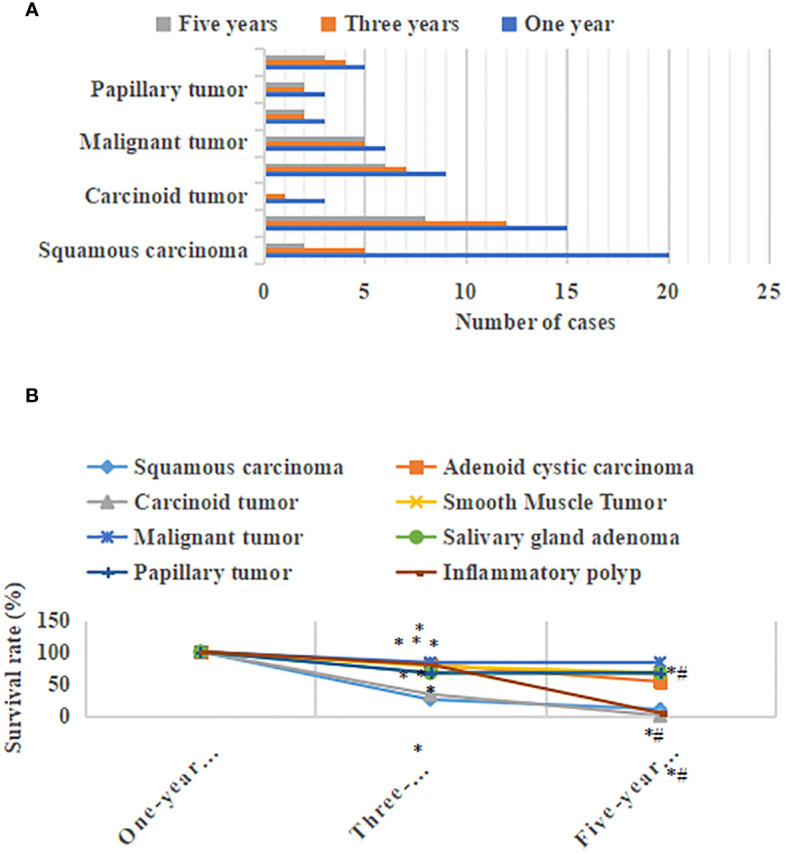
Postoperative survival of patients with different types of primary tracheal tumors. **(A)** postoperative survival distribution of the number of patients with different types of primary tracheal tumors; **(B)** distribution of postoperative survival rate of patients with different types of primary tracheal tumors * meant that compared with the one-year survival rate, P<0.05; *# meant that compared with the three-year survival rate, P<0.05.

**Figure 8** showed the postoperative survival of patients with different types of primary tracheal tumors. The three-year survival rate for patients with squamous carcinoma was 25%, and the five-year survival rate was 10%. Patients with adenoid cystic carcinoma had a three-year survival rate of 80% and a five-year survival rate of 53.3%. The 3-year survival rate for patients with carcinoid tumors was 33.3%, and the 5-year survival rate was 0% Patients with smooth muscle tumors had a 3-year survival rate of 77.8% and a 5-year survival rate of 66.7%. The 3-year and 5-year survival rates of the patients with malignant tumors were 83.3% and 83.3%, respectively. The 3-year survival rate of patients with salivary gland adenoma was 66.7%, and the 5-year survival rate was 66.7%. The 3-year survival rate of patients with papillary tumors was 66.7%, and the 5-year survival rate was 66.7%. Patients with inflammatory polyp had a three-year survival rate of 80% and a five-year survival rate of 80%. The 3-year and 5-year survival rates of patients with squamous carcinoma, adenoid cystic carcinoma, and carcinoid tumors were notably decreased, with a considerable difference (P<0.05). The three-year survival rate of patients with smooth muscle tumors, malignant tumors, salivary gland adenoma, papillary tumors, and the inflammatory polyp was markedly lower than that of the one-year survival rate, with a significant difference (P<0.05). There was an insignificant difference between the three-year survival rate and five-year survival rate (P>0.05).

### Incidence of postoperative complications and mortality

**Figure 9:**
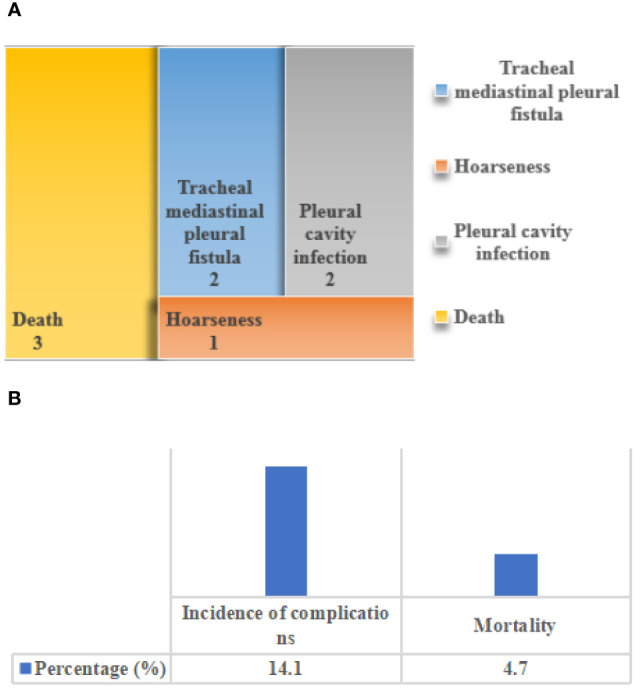
The incidence of postoperative complications and mortality of patients. **(A)** distribution of postoperative complications; **(B)** the incidence of postoperative complications and the distribution of mortality caused by complications.

**Figure 9** showed the incidence of postoperative complications and mortality of patients. Among the included 64 patients with primary tracheal tumors, 9 patients developed postoperative complications, including 2 cases of tracheal mediastinal pleural fistula, 2 cases of hoarseness, 1 case of pleural cavity infection, and 3 cases of death due to the complications. The incidence of postoperative complications was 14.1%, and the mortality rate due to complications was 4.7%.

## Discussion

At present, primary tracheal tumors are relatively rare, accounting for about 1% of respiratory tumors. According to the degree of differentiation, they can be classified into malignant, low malignant, and benign (22). Primary malignant tracheal tumors include squamous cell carcinoma, adenocarcinoma, and poorly differentiated carcinoma, among which squamous cell carcinoma is the most common that accounts for about 50% of primary tracheal tumors. The benign tumors include smooth muscle tumors, malignant tumors, papillary tumors, neurofibroma, mixed tumor of the salivary gland, and hemangioma. Moreover, there are also some rare tumors, such as carcinosarcoma, chondrosarcoma, and chondroma (23, 24). And some tracheal carcinomas may be misdiagnosed as asthma during routine examinations (25). Therefore, finding more accurate diagnostic methods is extremely important.

CT examination is a ubiquitous imaging diagnostic method for primary tracheal tumors in clinical practice. It can show the soft tissue image with increased density in the tracheal lumen, which is mostly eccentric, with the incrassation of the trachea wall and irregular tracheal stenosis. About 10% of tracheal tumors grow along the surrounding tracheal, while 30%-40% of tracheal tumors directly involve the mediastinum (26, 27). According to the CT images, the bronchial tumor grows into the lumen or infiltrate out of the lumen, which leads to bronchial insufficiency or complete obstruction as well as obstructive pneumonia or atelectasis based on the degree of infiltration (28). Multislice spiral CT can obtain the image data of multiple layers simultaneously because of its multi-row wide detector structure and single exposure of the bulb tube. Therefore, compared with ordinary CT, MSCT has such advantages as the shorter time consuming, greatly reduced radiation exposure of patients, more complete information collection, higher resolution, and clearer imaging. Furthermore, it is suitable for children and patients with severe illnesses (29). The results in this study are consistent with the previous period, and the accuracy of the images processed by MSCT in preoperative diagnosis is obviously improved.

Through the comparative analysis of MSCT images, primary tracheal tumors could be roughly classified into the intraluminal narrow basal nodule, the intraluminal wide basal nodule, infiltrative growth along the duct wall, intraluminal and extraluminal mass, and tracheal ridge mass (30, 31). The diagnostic accuracy of patients’ MSCT images reflected that, compared with original CT images, the diagnostic accuracy of the VR, MPVR, and CTVE images in tumor morphology, the extent of the long-axis involvement of the duct wall, and the degree of wall stenosis was obviously improved, with significant differences (P<0.05). MSCT imaging had a good practical value in the clinical diagnosis of primary tracheal tumors in cardiothoracic surgery, which was consistent with the research results of Tran et al. (2019) (32). In the study by Li et al., it was found that the use of MSCT could significantly identify the shape and density of duodenal adenomatous nodules, thereby improving the detection rate of tumors (32). Some research suggests that post-processing techniques of MSCT, such as multi-planar reconstruction, minimum density projection, volume rendering, and CT virtual endoscopy, can enhance the detection rate of duodenal tumors (TD) after CT technology and provide a clear display of their radiological characteristics (33). Additionally, studies have found significant differences in the 3-year survival rates among patients with different types of primary tracheal tumors. The 3-year survival rate of malignant tracheal tumor patients significantly decreases, while the 3-year survival rate of benign tumors remains relatively stable and with longer survival time. In Ran et al.’s study, it was found that primary tracheal adenoid cystic carcinoma had a metastasis rate of 24.9% postoperatively, with 5 and 10-year survival rates of 86.4% and 55.6%, respectively (34). Possibly due to sample differences, the 5-year survival rate of adenoid cystic carcinoma patients in this study was 53.3%. When exploring the safety of surgical treatment, it was found that the complications occurring after surgery were within clinically controllable ranges, and no serious clinical events occurred. The mortality rate due to complications was 4.7%. The three cases of primary tracheal tumor deaths caused by complications all had certain degrees of immune function issues. This indicates that surgical treatment demonstrates good therapeutic effects in the clinical management of primary tracheal tumors, greatly extending patient life and improving their quality of life (35).

## Conclusion

The MSCT image features of patients with different types of primary tracheal tumors were innovatively analyzed, and different MSCT image post-processing methods were compared and analyzed. Besides, the results of surgical treatment for the disease were quantified. The results showed that compared with original CT, the diagnostic accuracy of the MSCT on tumor morphology, the involvement range of the long axis of the tube wall, and the degree of lumen stenosis in patients with primary tracheal tumor was obviously improved. After surgical treatment of primary tracheal tumors, the 3-year survival rate and 5-year survival rate of malignant tumors decreased markedly in contrast to the 1-year survival rate. There was an insignificant difference in the 3-year survival rate and 5-year survival rate for benign tumors. In conclusion, MSCT imaging had a good performance in the diagnosis of primary tracheal tumors, and surgical treatment had a substantial effect on primary tracheal tumors, which was worthy of further promotion.

## Data availability statement

The original contributions presented in the study are included in the article/supplementary material. Further inquiries can be directed to the corresponding author.

## Ethics statement

This study was conducted according to the guidelines laid down in the Declaration of Helsinki and all procedures involving research study participants were outlined in the study protocols approved by the Wuxi Second People’s Hospital and was conducted in agreement with principles of Helsinki declarations and local ethical standards. Patients of study participants provided written informed consent.

## Author contributions

JR: Writing – review & editing, Supervision, Methodology, Data curation. YL: Writing – original draft, Software, Investigation, Conceptualization.
